# Evaluation of Conjunctival Autografting Augmented with Mitomycin C Application *versus* Ologen Implantation in the Surgical Treatment of Recurrent Pterygium

**DOI:** 10.1155/2021/8820926

**Published:** 2021-01-12

**Authors:** Faried M. Wagdy, Hassan G. Farahat, Amin F. Ellakwa, Sameh S. Mandour

**Affiliations:** Department of Ophthalmology, Faculty of Medicine, Menofia University, Shebin El Kom, Menofia, Egypt

## Abstract

**Objectives:**

To evaluate the safety and efficacy of augmenting conjunctival autografting with intraoperative mitomycin C (MMC) application *versus* Ologen implantation in the management of recurrent pterygium.

**Materials and Methods:**

This prospective randomised study included 63 eyes of 63 patients, with recurrent nasal pterygium, who presented to the outpatient clinic of Menoufia University Hospital in Shebin El Kom and Manshiet Soltan from January 2016 to December 2019. Patients were randomly enrolled into two groups. Group A included 32 eyes of 32 patients who underwent conjunctival autografting augmented with the topical application of MMC (0.2 mg/mL), and group B included 31 eyes of 31 patients who underwent conjunctival autografting augmented with Ologen implantation. All the patients underwent follow-up examinations for a period of 24 months. During each visit, a complete ophthalmic examination was performed. Pterygium regrowth of 1 mm or more, over the cornea, was considered a recurrence.

**Results:**

In the MMC group, no recurrence was reported during the 24-month follow-up period. In the Ologen implantation group, recurrence was reported in 2 (8%) eyes. The time interval from surgery to recurrence was 5 months in one case and 8 months in the other. No other serious postoperative complications were reported, and there was no statistically significant difference between the groups in this regard.

**Conclusion:**

Ologen implantation with conjunctival autografting shows promising results in the surgical management of recurrent pterygium with mild non-vision-threatening postoperative complications comparable to that of MMC application with conjunctival autografting. Registration number: *ClinicalTrials.gov*NCT04419038.

## 1. Introduction

The tendency of pterygia to recur after surgical removal is frustrating for both patients and surgeons [1]. Most ophthalmologists define pterygium recurrence as corneal recurrence, which includes a regrowth of the fibrovascular pterygium-like tissue crossing the limbus onto the cornea, fibrovascular recurrence attaining the same degree of corneal encroachment as the original lesion, or a regrowth exceeding 1 mm onto the cornea [2].

The risk factors for recurrence include male sex and a history of high sun exposure [3]. Nontranslucent pterygia have been associated with an increased risk of recurrence, as they have several biomarkers related to proliferation, inflammation, fibrosis, and angiogenesis. While the further evaluation of these important biomarkers is needed, they point out the need to develop treatments that target specific receptors in order to achieve the goal of eliminating recurrence [4].

Several surgical techniques and modifications have been used to treat recurrent pterygia. However, none of them have been proven to be completely effective in eliminating further recurrence. A combination of more than one method may usually yield promising results in this regard. Antimetabolites, such as mitomycin C (MMC), and antifibrotic agents, such as Ologen, have been used as adjuvants by many investigators to reduce the possibility of recurrence [5, 6].

This study aimed to evaluate the safety and efficacy of augmenting conjunctival autografting with intraoperative MMC application *versus* Ologen implantation in the management of recurrent pterygium.

## 2. Materials and Methods

This was a prospective randomised study that included 63 eyes of 63 patients with recurrent nasal pterygium who presented to the outpatient clinic of Menoufia University Hospital in Shebin El Kom and Manshiet Soltan during the period from January 2016 to December 2019

Patients were randomly enrolled into two groups. Group A included 32 eyes of 32 patients who underwent conjunctival autografting augmented with the topical application of MMC (0.2 mg/mL). Group B included 31 eyes of 31 patients who underwent conjunctival autografting augmented with Ologen implantation.

All patients had a recurrent nasal pterygium after one surgical session for the removal of the primary pterygium. Patients with a primary nasal pterygium, or a recurrent pterygium, who underwent more than one session for the surgical removal were excluded. Patients with cicatrising conjunctival disease or previous conjunctival surgery were also excluded from the study.

A comprehensive ophthalmic examination, including best-corrected visual acuity testing, slit-lamp examination, Goldmann applanation tonometry (whenever possible), fundus examination, and ocular motility examination, was performed for all patients. According to their corneal extent, the pterygia recurrences were classified into 3 grades:

  Grade 1: fibrovascular proliferations extend up to one-quarter of the corneal diameter.
  Grade 2: fibrovascular proliferations extend up to the centre of the cornea.
  Grade 3: fibrovascular proliferations extend beyond the visual axis.

Informed consent was obtained from all patients, and the study was approved by the institutional review board. All measures were in accordance with the tenets of the Declaration of Helsinki.

As for group A, 0.2 mg/mL MMC was prepared by reconstituting a 2 mg vial of mitomycin with 10 mL of sterile water for injection. When using a 10 mg vial of mitomycin, the same concentration was achieved by first reconstituting it with 10 mL of sterile water and then diluting 1 mL of the prepared solution with 4 mL of saline to achieve a concentration of 0.2 mg/mL (0.02% solution). This solution was stable for two weeks under refrigeration and for 24 h at room temperature (59–86°F).

As for group B, half disc of porous collagen matrix (Ologen™; Aeon Astron Europe B.V., The Netherlands) was used for each patient. The Ologen disc was 6 mm in diameter and 2 mm thick.

## 3. Operative Procedure

**Figure 1:**
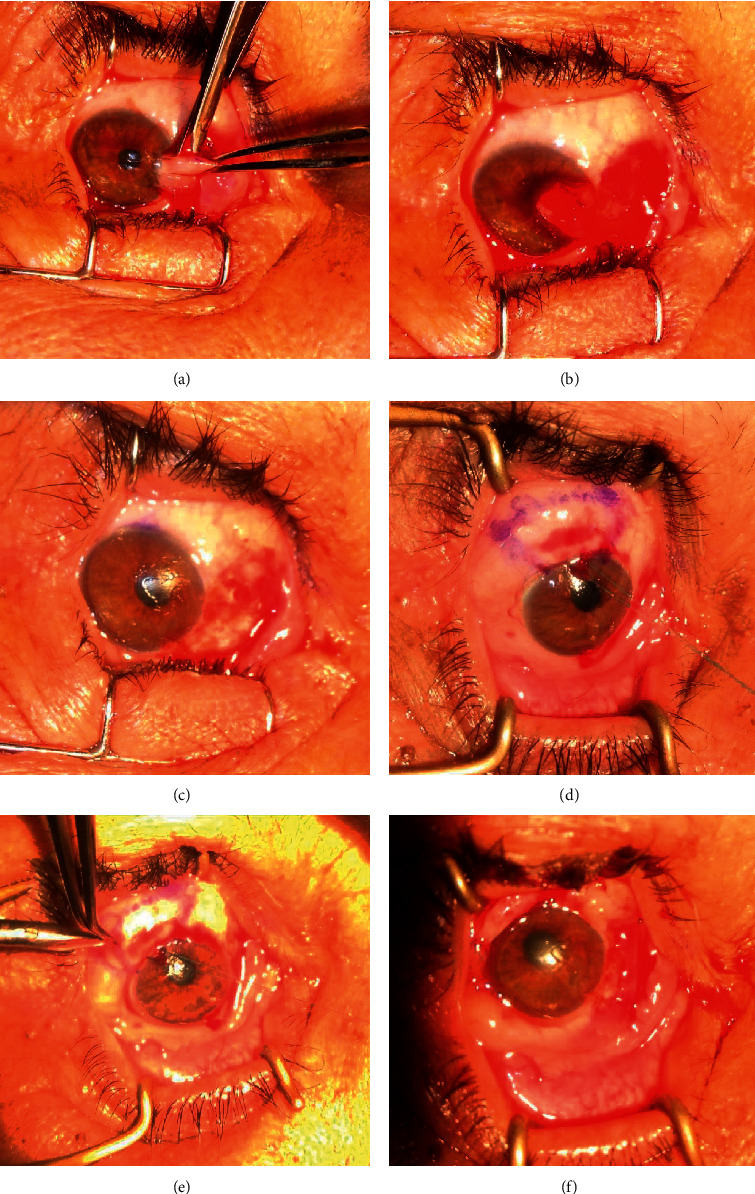
Basic operative technique: (a) dissection of the pterygium from the scleral bed; (b) complete separation of body of pterygium from the scleral bed; (c) bare sclera and cornea after pterygium excision; (d) marking of the conjunctival autograft; (e) dissection of the conjunctival autograft from its bed; (f) suture of the graft to the bare sclera.

All patients underwent surgery under peribulbar anaesthesia. Two radial incisions were made in the conjunctiva and Tenon's capsule at the upper and lower limits of the pterygium belly from the limbus to approximately 4-5 mm towards the inner canthus. The belly was then carefully dissected from the underlying sclera (Figure 1(a)).

The head of the pterygium was dissected from its corneal attachment by reverse stripping using slow and deliberate traction, holding its free end parallel to the cornea (Figure 1(b)).

The fibrovascular tissue underneath the cut end of the conjunctiva was dissected as far as possible on the canthus side and excised, leaving the sclera and medial rectus muscle free from episcleral tissue. Cautery was applied to the bare sclera to control bleeding (Figure 1(c)).

In group A, MMC (0.02%) was applied using a soaked sponge of 3 × 4 mm to the bare sclera area for 2 min, followed by copious irrigation with normal saline. Donor tissue was harvested from the superotemporal conjunctiva of the same eye. The eyeball was rotated down using a superior limbal suture. An area of the conjunctiva larger than the bare sclera area by 1 mm was measured with callipers and marked with gentian violet (Figure 1(d)). The conjunctiva was elevated with the subconjunctival injection of lidocaine 2% and epinephrine 1 : 100,000.

Conjunctival scissors were used to make two parallel radial incisions along the marked lines and to undermine the conjunctiva along the lateral borders (Figure 1(e)). When the posterior and lateral ends of the graft were free, blunt dissection was continued anteriorly until the limbus, and a crescent knife or No. 15 blade was used to carry out a further blunt dissection towards the cornea. This dissection continued into the peripheral cornea approximately 1 mm beyond the vascular arcade. The conjunctival piece was excised using sharp Vannas scissors. The graft was transferred to the bare sclera, epithelial side up, without losing the limbal orientation (the limbal border was identified by its brownish colour that was tough and firm, somewhat characterised by a wavy margin). The epithelial surface was recognised from the stromal side by its smooth and shiny surface, and it was slippery in nature without adherence to the wicks.

The four corners of the graft were then secured using 10-0 polyamide interrupted sutures (Figure 1(f)).

**Figure 2:**
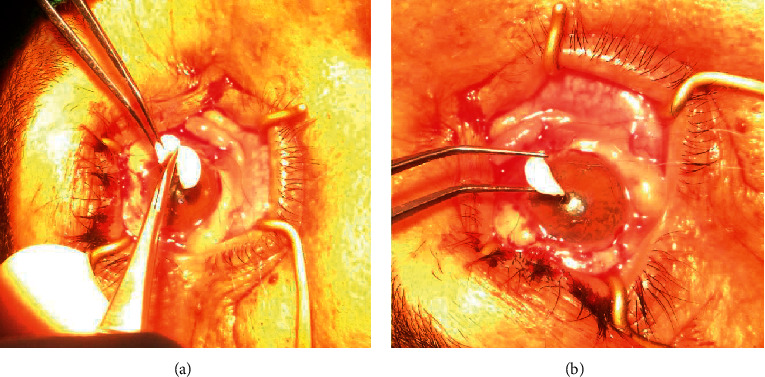
Ologen implantation: (a) cutting Ologen disc into two halves; (b) implanting Ologen under the graft before securing it in place.

In group B, Ologen was applied under the graft tissue once it was secured in place (half the vial was used for every patient) (Figures 2(a) and 2(b)). Multiple 10-0 polyamide interrupted sutures were used to fix the graft. The donor site was left open for spontaneous healing.

All patients were re-examined at one week, one month, three months, and once every three months thereafter, for a total period of 24 months. During each visit, a complete ophthalmic examination was performed. A pterygium regrowth over the cornea of 1 mm or more was considered a recurrence.

Recorded data were analysed using the Statistical Package for Social Sciences, version 16.0 (SPSS Inc., Chicago, Illinois, USA). Quantitative data were expressed as mean ± standard deviation (SD), and significance was determined using an independent sample *t-test*. Qualitative data were expressed as frequency and percentage, and significance was tested using a chi square test, with the level of significance set at 95%.

## 4. Results

Sixty-three eyes of 63 patients were enrolled in the study, including 32 eyes in group A and 31 eyes in group B. There were 21 (58.3%) men and 11 (40.7%) women in group A and 15 (48.4%) men and 16 (51.6%) women in group B, as shown in Table 1. The mean age of patients in group A was 41.8 ± 15.98 years, while the mean age in group B was 46.2 ± 12.77 years, with no statistically significant difference between both groups regarding gender and age distribution.

The distribution of the three preoperative clinical grades of pterygia in both groups is shown in Table 2, and the distribution of the indication for surgery in each group is shown in Table 3. The difference between both groups regarding both parameters was statistically insignificant.

Table 4 shows the mean preoperative best-corrected visual acuity (BCVA) as well as the mean postoperative BCVA at the end of the follow-up period in both groups. Mean postoperative BCVA improved; however, there was no statistically significant difference regarding this improvement between both groups.

Regarding postoperative complications, the most important one was the recurrence rate. In group A, no reported recurrence was found during the 24-month follow-up period. In group B, a recurrence was reported in 2 eyes (8%). The time interval from surgery to recurrence was 5 months in one case and 8 months in the other. There was no statistically significant difference between both groups regarding postoperative recurrence (*P*=0.197).

Other postoperative complications are summarised in Table 5. No serious postoperative complications were reported. There was no statistically significant difference between the groups in this regard as well.

The two recurrent cases in group B refused further surgery. Their recurrent pterygium was still off centre, and they are still being followed up. The haematomas under the graft were drained through a 1 mm incision in the graft on the first postoperative day. The granulomas were excised if they were resistant to postoperative medical treatment.

## 5. Discussion

Pterygium is a common eye disease worldwide, especially in tropical and subtropical areas [7]. It is only treated by surgical removal when it affects vision or cosmesis. However, recurrence is the most common postoperative complication. This poses a challenging situation that may affect the integrity and health of the ocular surface in the long run. Recurrent pterygia differ histopathologically from primary pterygia, have higher rates of recurrence with subsequent surgical interventions, and must be considered a different entity from primary pterygia. Recurrent pterygia are also considered to be clinically more aggressive and therefore are more likely to cause visual deficits and other complications [8].

To effectively treat recurrent pterygium, a good understanding of its pathogenesis is crucial. Pterygium is not just a degenerative lesion but could also occur as a result of uncontrolled cell proliferation. Matrix metalloproteinases (MMPs) and tissue inhibitors of MMPs (TIMPs) at the advancing pterygium edge may be responsible for the inflammation, tissue remodelling, and angiogenesis that characterize pterygia, as well as the destruction of Bowman's layer and invasion of the pterygium into the cornea. High levels of vascular endothelial growth factor (VEGF) as well as inflammatory cytokines and mediators were detected in pterygial tissues, relative to normal conjunctiva. It has also been speculated that pterygium may represent an area of localized limbal stem cell deficiency [2, 4].

Pterygium excision and conjunctival limbal autograft is considered the gold standard and is currently one of the most commonly used methods for pterygium surgery because of the reported low recurrence rates. However, this method generally results in higher recurrence rates when used for treatment of recurrent pterygium, ranging from 11.3% to 50% [9–14]. Recurrence can occur as a result of inadequate peripheral dissection, insufficient graft size, thick graft with Tenon tissue, and graft retraction due to inadequate fixation [8]. For this reason, and to avoid further recurrence, it is necessary to augment the effect of conjunctival autograft. Adding an adjuvant that acts by decreasing fibrosis would offer the required benefit. Hence, in the current study, the authors combined conjunctival autograft with the intraoperative use of MMC in one group and Ologen implantation in the other.

MMC, an alkylating agent that reduces recurrence by inhibiting fibrovascular growth, has been shown to be a useful adjunct to pterygium surgery [5, 10, 15–17]. An intraoperative topical application of a low-concentration MMC (0.02% for 2 min) was shown to be effective in reducing recurrence with minimal risk of serious complications such as scleral melting [18]. Segev et al. reported an even lower recurrence rate of 2% in a subgroup of patients with recurrent pterygium using MMC 0.02% for 2 min and conjunctival graft without serious complications [19]. Considering the safety and efficacy of the combined method using conjunctival autograft and MMC, we used this method in cases of recurrent pterygium.

In the current study, no sight-threatening complications were reported in the MMC group. Moreover, no recurrence was reported during the 24-month follow-up period in this group. However, several studies indicate that severe complications can occur months after the initial treatment; the complications include cataract formation, anterior uveitis, scleral plaque and necrosis, corneal oedema and ulceration, protracted pain, anterior chamber inflammation and nonhealing conjunctival, and corneal and scleral defects [8].

Ologen, a bioengineered, biodegradable, porous collagen-glycosaminoglycan matrix implant, has been developed as an adjuvant to decrease scarring in glaucoma surgery. Ologen reduces conjunctival contraction and promotes the formation of an almost normal subconjunctival stroma [20]. In addition, the matrix improves regenerating tissue remodelling and prevents scar formation or further infection [21].

Ologen was used successfully to minimise scarring in subscleral trabeculectomy [22] and dacryocystorhinostomy [23] and to prevent recurrence after primary pterygium removal [24, 25]. Therefore, the authors in the current study used Ologen as an augmenting agent with conjunctival autografting for recurrent pterygium in the second group of patients. The authors in the current work reported 2 cases of recurrence in this group during the 24-month follow-up period. However, there was no statistically significant difference in recurrence rate between this group and the MMC group.

Other postoperative complications in the current study included haematoma under the graft, granuloma formation, infection, suture-related inflammation, and inclusion cyst and graft invasion or retraction, which were vision-non-threatening. There was no statistically significant difference between both groups in this regard. This may suggest that it is advantageous to use Ologen as a substitute for MMC, as the topical application of the latter has raised a major concern regarding sight-threatening complications that may even appear later in time [26, 27].

However, there were some pitfalls in this study, specifically limiting the candidate patients to those who had only one recurrence episode. The authors plan to apply the same study design and techniques in the treatment of patients with more than one recurrence. The limited number of those patients may mean that more time will be required to recruit enough patients to obtain statistically significant results.

## 6. Conclusion

Ologen implantation with conjunctival autografting shows promising results in the surgical management of recurrent pterygium, comparable to those of MMC application combined to conjunctival autografting, with mild non-vision-threatening postoperative complications.

## Figures and Tables

**Table 1:** Distribution of patients in both groups according to their gender.

Variable	Methods				*x* ^2^	*P*
	Mitomycin C		Ologen			
	No.	%	No.	%		
Sex					**1.9**	**0.17**
Male	21	58.3	15	48.4		
Female	11	40.7	16	51.6		
Total	**32**	**100.0**	**31**	**100.0**		

**Table 2:** Distribution of the clinical grades of pterygia in both groups.

Variable	Methods				*x* ^2^	*P*
	Mitomycin C		Ologen			
	No.	%	No.	%		
Grade					**0.397**	**0.82**
Grade I	8	25.0	9	29.0		
Grade II	20	62.5	17	54.8		
Grade III	4	12.5	5	16.2		
Total	**32**	**100.0**	**25**	**100.0**		

**Table 3:** Distribution of patients in both groups regarding the indications of surgery.

Variable	Methods				*x* ^2^	*P*
	Mitomycin C		Ologen			
	No.	%	No.	%		
Indications for surgery						
Invading or threatening visual axis	4	12.5	5	16.1		
Visual impairment due to astigmatism	11	34.4	7	22.6	1.17	0.67
Irritative symptoms and inflammation	10	31.2	12	38.7		
Cosmesis	7	21.9	7	22.6		
**Total**	**32**	**100.0**	**31**	**100.0**		

**Table 4:** Comparison between the two groups regarding pre- and postoperative BCVA.

Variable	Methods		*P*
	Mitomycin C	Ologen	
Preoperative BCVA	0.68 ± 0.22	0.73 ± 0.21	0.41
Postoperative BCVA	0.89 ± 0.12	0.87 ± 0.19	0.57

**Table 5:** Comparison between the two groups in relation to postoperative complications.

Variable	Methods				*x* ^2^	*P*
	Mitomycin C		Ologen			
	No.	%	No.	%		
Postoperative complications						
None	25	78.1	19	61.3	8.338	0.304
Haematoma under graft	2	6.2	2	6.5		
Granuloma formation	0	0.0	1	3.2		
Infection	2	6.2	1	3.2		
Recurrence	**0**	**0.0**	**2**	**6.5**		
Suture-related inflammation	**2**	6.2	**3**	**9.7**		
Inclusion cyst	**1**	3.1	**0**	**0.0**		
Graft invasion or retraction	**0**	**0.0**	**3**	**9.7**		
Total	**32**	**100.0**	**31**	**100.0**		

## Data Availability

Data used to support the findings of this study are available on request from the corresponding author.
